# Reframing climate change as a public health issue: an exploratory study of public reactions

**DOI:** 10.1186/1471-2458-10-299

**Published:** 2010-06-01

**Authors:** Edward W Maibach, Matthew Nisbet, Paula Baldwin, Karen Akerlof, Guoqing Diao

**Affiliations:** 1 Center for Climate Change Communication, Department of Communication, George Mason University, Fairfax, VA, USA; 2 School of Communication, American University, Washington, DC, USA; 3 Department of Statistics, George Mason University, Fairfax, VA, USA

## Abstract

**Background:**

Climate change is taking a toll on human health, and some leaders in the public health community have urged their colleagues to give voice to its health implications. Previous research has shown that Americans are only dimly aware of the health implications of climate change, yet the literature on issue framing suggests that providing a novel frame - such as human health - may be potentially useful in enhancing public engagement. We conducted an exploratory study in the United States of people's reactions to a public health-framed short essay on climate change.

**Methods:**

U.S. adult respondents (n = 70), stratified by six previously identified audience segments, read the essay and were asked to highlight in green or pink any portions of the essay they found "especially clear and helpful" or alternatively "especially confusing or unhelpful." Two dependent measures were created: a composite sentence-specific score based on reactions to all 18 sentences in the essay; and respondents' general reactions to the essay that were coded for valence (positive, neutral, or negative). We tested the hypothesis that five of the six audience segments would respond positively to the essay on both dependent measures.

**Results:**

There was clear evidence that two of the five segments responded positively to the public health essay, and mixed evidence that two other responded positively. There was limited evidence that the fifth segment responded positively. Post-hoc analysis showed that five of the six segments responded more positively to information about the health benefits associated with mitigation-related policy actions than to information about the health risks of climate change.

**Conclusions:**

Presentations about climate change that encourage people to consider its human health relevance appear likely to provide many Americans with a useful and engaging new frame of reference. Information about the potential health benefits of specific mitigation-related policy actions appears to be particularly compelling. We believe that the public health community has an important perspective to share about climate change, a perspective that makes the problem more personally relevant, significant, and understandable to members of the public.

## Background

Climate change is already taking a toll on human health in the United States [1] and other nations worldwide [2]. Unless greenhouse gas emissions worldwide are sharply curtailed - and significant actions taken to help communities adapt to changes in their climate that are unavoidable - the human toll of climate change is likely to become dramatically worse over the next several decades and beyond [3]. Globally, the human health impacts of climate change will continue to differentially affect the world's poorest nations, where populations endemically suffer myriad health burdens associated with extreme poverty that are being exacerbated by the changing climate. As stated in a recent *British Medical Journal *editorial, failure of the world's nations to successfully curtail emissions will likely lead to a "global health catastrophe" [4]. In developed countries such as the United States, the segments of the population most at risk are the poor, the very young, the elderly, those already in poor health, the disabled, individuals living alone, those with inadequate housing or basic services, and/or individuals who lack access to affordable health care or who live in areas with weak public health systems. These population segments disproportionately include racial, ethnic, and indigenous minorities [5].

While legislation to reduce U.S. greenhouse gas (GHG) emissions has stalled in Congress, in December 2009 the Environmental Protection Agency (EPA) moved toward regulating carbon dioxide and five other of the gases under the Clean Air Act, citing its authority to protect public health and welfare from the impacts of global warming [5]. The agency found that global warming poses public health risks - including increased morbidity and mortality - due to declining air quality, rising temperatures, increased frequency of extreme weather events, and higher incidences of food- and water-borne pathogens and allergens.

This finding comes as a relatively small group of public health professionals are working rapidly to better comprehend and quantify the nature and magnitude of these threats to human health and wellbeing [6]. This new but rapidly advancing public health focus has received minimal news media attention, even at internationally leading news organizations such as the *New York Times *[unpublished data]. It is not surprising therefore that the public also has yet to fully comprehend the public health implications of climate change. Recent surveys of Americans [7], Canadians [8], and Maltese [9] demonstrate that the human health consequences of climate change are seriously underestimated and/or poorly understood, if grasped at all. About half of American survey respondents, for example, selected "don't know" (rather than "none," "hundreds," "thousands," or "millions") when asked the estimated number of current and future (i.e. 50 years hence) injuries and illnesses, and death due to climate change. An earlier survey of Americans [10] demonstrated that most people see climate change as a geographically and temporally distant threat to the non-human environment. Notably, not a single survey respondent freely associated climate change as representing a threat to people. Similarly, few Canadians, without prompting, can name any specific human health threat linked to climate change impacts in their country [8].

Cognitive research over the past several decades has shown that how people "frame" an issue - i.e., how they mentally organize and discuss with others the issue's central ideas - greatly influences how they understand the nature of the problem, who or what they see as being responsible for the problem, and what they feel should be done to address the problem [11,12]. The polling data cited above [7-9] suggests that the dominant mental frame used by most members of the public to organize their conceptions about climate change is that of "climate change as an environmental problem." However, when climate change is framed as an environmental problem, this interpretation likely distances many people from the issue and contributes to a lack of serious and sustained public engagement necessary to develop solutions. This focus is also susceptible to a dominant counter frame that the best solution is to continue to grow the economy - paying for adaptive measures in the future when, theoretically, society will be wealthier and better able to afford them - rather than focus on the root causes of the environmental problem [13]. This economic frame likely leaves the public ambivalent about policy action and works to the advantage of industries that are reluctant to reduce their carbon intensity. Indeed, it is precisely the lack of a countervailing populist movement on climate change that has made policy solutions so difficult to enact [13,14].

Significant efforts have been made over the past several years by public health organizations to raise awareness of the public health implications of climate change and prepare the public health workforce to respond, although as noted above, it is not clear the extent to which public health professionals, journalists, or most importantly, the public and policy makers have taken notice. In the United States, National Public Health Week 2008 was themed "Climate Change: Our Health in the Balance," the Centers for Disease Control and Prevention created a Climate Change and Public Health program, and several professional associations assessed the public health system's readiness to respond to the emerging threat [15-17]. Globally, World Health Day 2008 was themed "Protecting Health from Climate Change," and the World Health Organization has developed a climate change and health work plan, the first objective of which is "raising awareness of the effects of climate change on health, in order to prompt action for public health measures" [18]. Several prominent medical journals have released special issues on climate change and health [19-21], and these and other medical journals [4] have issued strongly worded editorials urging health professionals to give voice to the health implications of climate change.

An important assumption in these calls to action is that there may be considerable value in introducing a public health frame into the ongoing public - and policy - dialogue about climate change. While there is indeed solid theoretical basis for this assumption, to the best of our knowledge there is not yet empirical evidence to support the validity of the assumption [22].

The purpose of this study therefore was to explore how American adults respond to an essay about climate change framed as a public health issue. Our hypothesis was that a public health-framed explanation of climate change would be perceived as useful and personally relevant by readers, with the exception of members of one small segment of Americans who dismiss the notion that human-induced climate change is happening. We used two dependent measures in this hypothesis: a composite score based on respondent reactions to each sentence in the essay, and the overall valence of respondents' general comments made after reading the essay.

**Figure 1:**
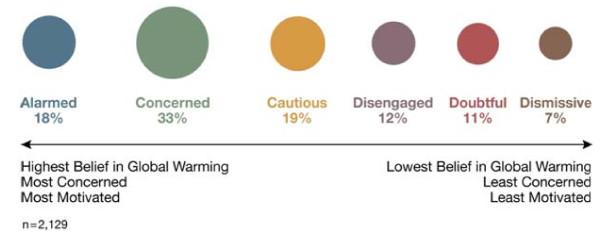
**Global Warming's Six Americas**. A nationally representative sample of American adults classified into six unique audience segments based on their climate change-related beliefs, behaviors and policy preferences.

Our study builds on previous research that identified six distinct segments of Americans, termed *Global Warming's Six Americas *[7]. These six segments of Americans - the Alarmed (18% of the adult population), the Concerned (33%), the Cautious (19%), the Disengaged (12%), the Doubtful (11%), and the Dismissive (7%) - fall along a continuum from those who are engaged on the issue and looking for ways to take appropriate actions (the Alarmed) to those who actively deny its reality and are looking for ways to oppose societal action (the Dismissive; see Figure 1). The four segments in the middle of the continuum are likely to benefit most from a reframing of climate change as a human health problem because, to a greater or lesser degree, they are not yet sure that they fully understand the issue and are still, if motivated to do so, relatively open to learning about new perspectives.

## Methods

### Sample

Between May and August 2009, 74 adults were recruited to participate in semi-structured in-depth elicitation interviews that lasted an average of 43 minutes (ranging from 16 to 124 minutes) and included the presentation of a public health framed essay on climate change. The recruitment process was designed to yield completed interviews with a demographically and geographically diverse group of at least 10 people from each of the previously identified "Six Americas" [7]. Four respondents were dropped from this study due to incomplete data, leaving a sample size of 70. Audience segment status (i.e., which one of the "Six Americas" a person belonged) was assessed with a previously developed 15-item screening questionnaire that identifies segment status with 80% accuracy [unpublished data].

To achieve demographic diversity in the sample, we recruited an approximately balanced number of men and women, and an approximately balanced number of younger (18 to 30), middle-aged (31 to 50), and older (51 and older) adults (see Table 1). We did not set recruitment quotas for racial/ethnic groups, but did make an effort to recruit a mix of people from various racial/ethnic backgrounds.

**Table 1:** Distribution of Respondents by Age, Gender and Segment.

Gender	Age	Alarmed	Concerned	Cautious
Female	18 - 30	2	3	1

	31 - 50	2	1	2

	50+	2	3	2

Male	18 - 30	1	2	3

	31 - 50	2	1	3

	50+	2	3	2

Total		11	13	13

				

**Gender**	**Age**	**Disengaged**	**Doubtful**	**Dismissive**

Female	18 - 30	3	2	2

	31 - 50	2	1	1

	50+	0	1	1

Male	18 - 30	1	2	2

	31 - 50	2	4	3

	50+	2	2	2

Total		10	12	11

To achieve geographic diversity, we recruited participants in one of two ways. The majority of participants (n = 56) were recruited - and then interviewed - face-to-face in one of two locations: out-of-town visitors were interviewed at a central location on the National Mall in Washington, DC (a national park situated between the U.S. Capitol, the Smithsonian Museum buildings, and the Lincoln Memorial); and shoppers were interviewed at an "outlet" mall (i.e., discount branded merchandise shopping mall) adjacent to an interstate freeway in Hagerstown, MD. The outlet mall is more than an hour driving distance outside of Washington, DC and attracts shoppers from Maryland, Pennsylvania, and West Virginia, as well as visitors from further away who are driving the interstate freeway. The remaining study participants were recruited via email from among participants to a nationally representative survey that we conducted in Fall 2008 [7]. They were interviewed subsequently by telephone, after being mailed a copy of the test "public health essay" - described below - in a sealed envelope marked "do not open until asked to do so by the interviewer." As an incentive to participate, all respondents were given a $50 gift card upon completion of their interview. George Mason University Human Subjects Review Board provided approval for the study protocol (reference #6161); all potential respondents received written consent information prior to participation.

The 70 study participants resided in 29 states. Using U.S. Census Bureau classifications, 14% (n = 10) were from the Northeast region, 21% (n = 15) were from the Midwest, 40% (n = 28) from the South, and 23% (n = 16) were from the West; state and region were unknown for one participant. In 2006, the geographic distribution of the overall U.S. population was 18%, 22%, 36% and 23% in the Northeast, Midwest, South and West, respectively [23].

### Data Collection and Coding

The majority of the interview was devoted to open-ended questions intended to establish the respondent's emotions, attitudes, beliefs, knowledge and behavior relative to global warming's causes and consequences. For example, respective open-ended questions asked alternatively if, how, and for whom global warming was a problem; how global warming is caused; if and how global warming can be stopped or limited; and what, if anything, an individual could do to help limit global warming. Toward the end of the interview, respondents were asked to read "a brief essay about global warming" (see Appendix 1), which was designed to frame climate change as a human health issue. Respondents were also given a green and a pink highlighting pen and asked to "use the green highlighter pen to mark any portions of the essay that you feel are especially clear or helpful, and use the pink highlighter pen to mark any portions of the essay that are particularly confusing or unhelpful."

As shown in Appendix 1, the one page essay was organized into four sections: an opening paragraph that introduced the public health frame (5 total sentences); a paragraph that emphasized how human health will be harmed if action is not taken to stop, limit, and/or protect against global warming (i.e., a description of the threat; 7 sentences); a paragraph that discussed several mitigation-focused policy actions and their human health-related benefits if adopted (4 sentences); and a brief concluding paragraph intended to reinforce the public health frame (2 sentences).

When respondents finished the reading, they were asked to describe in an open-ended format their "general reaction to this essay." (Note: This question was inadvertently not asked of one respondent, therefore the sample size for analysis of this data is 69.) For each portion of the essay they marked in green, they were subsequently asked: "What about each of these sentences was especially clear or helpful to you?" For each portion of the essay they marked in pink, they were also asked: "What about each of these sentences was especially confusing or unhelpful to you?"

To evaluate the respondent's general reactions to the essay we reviewed their individual statements (n = 193), defined as discrete thoughts or concepts. Based on this review, we iteratively developed eight thematic categories that captured the range of statements made by respondents. Table 2 defines and describe these themes.

**Table 2:** Thematic Categories Used to Code Respondents' General Reactions to the Public Health Essay.

Theme	Description
Lack of Evidence and/or Stylistically Confusing (Critique of Proof/Style)	Remarks indicate that adequate evidence was not given to support the arguments made (e.g., "it needs to include references to studies ... instead of just making ... these general statements") or that the essay was written poorly or was confusing (e.g., "I kind of see what they're saying, but to me it seems a little off-track with the rest of the essay").

Reflects Personal Point of View (Reflects My POV)	Remarks indicate agreement with the statement(s) in the essay (e.g., "... it captures what I believe," or "I strongly agree with this essay").

Informative and/or Thought-Provoking (Informative)	Remarks indicate that valuable information was provided (e.g., "It's informative, a lot of things I didn't know relate to global warming") or the essay sparked some self-reflexive thought processes (e.g., "It kind of opened my eyes up ...").

Biased and/or Alarmist (Biased)	Remarks indicate that the essay was written from a biased point of view or that the intention of the essay was to unjustly alarm the reader, (e.g., "There's an agenda ... to promote the junk science of global warming" or "It felt like scare tactics").

Evoked Negative Emotion, Fear, or Despair (Negative)	Remarks indicate the essay prompted negative feelings such as despair, lack of hope, fear, depression, or alarm.

Prescriptive	Remarks indicate useful information was provided on how to counter global warming (e.g., "... it's a good summation of how we should direct our research and direct our habits ..." or "it focused on how we can take action to make positive change").

Establishes Credibility (Credible)	Remarks express that the essay established credibility by providing specific examples such as West Nile virus or by referencing expert sources and authorities such as the World Health Organization or *The Lancet*.

American-centric or Too Closely Focused on the U.S. Perspective (Too U.S. Centric)	Remarks indicate that the essay focused too much on the United States with not enough of a global focus (e.g., "I felt they left out the world in general and focused specifically on just America ... it's not just the United States that needs to make changes").

Not Applicable (NA)	Remarks lacked relevant content or fell outside any of the above themes.

Two graduate student coders were then trained to code each statement into one of the thematic categories. The coders were also instructed to assess the overall valence of each respondent's statements - the first of our dependent measures - rating them as: -1 (entirely negative comments); 0 (mixed, including both positive and negative comments); or 1 (entirely positive comments). Following standard content analysis procedures, we tested inter-coder agreement on approximately 50 statements, making sure that a full range of possible types of coding decisions were required of the coders. To assess reliability, we used Krippendorff's alpha [24,25], a conservative measure that corrects for chance agreement among coders; a K-alpha of .70 or higher is considered sufficient and .80 or higher is considered excellent. For 7 of the 8 thematic categories, we achieved a reliability of .80 or higher; "Lack of Evidence or Stylistically Confusing" was the exception, with an inter-coder reliability of .70. After establishing reliability, the two coders then went on to categorize the rest of the remaining statements from the sample of respondents.

To code the respondent's sentence-specific reactions made with the highlighting pens, sentences marked with only green on at least one word were scored +1 (i.e. indicating "especially clear or useful"), sentences marked with only pink on at least one word were scored -1 (i.e. indicating "especially confusing or unhelpful), and sentences with either no highlighting, or both green and pink, were scored 0. Composite scores were created for each of the four sections of the essay - the opening, the threat section, the benefit section, and the conclusion - by summing the sentence-specific scores in the section and dividing by the number of sentences. A composite score for the entire essay - the second of the dependent measures in our hypothesis - was created by summing the sentence scores across each segment and dividing by the number of respondents per segment. Population estimates, which can be taken solely as preliminary indicators given the non-probabilistic nature of our sampling, were estimated by weighting the mean values for each of the six segments according to its prevalence in the U.S. population (see Figure 1).

### Data Analysis

**Figure 2:**
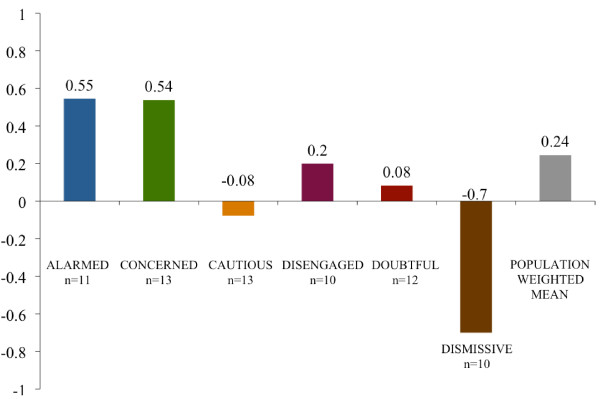
**Average valence of respondents' general essay comments**. The mean valence of respondent comments when asked their general reactions to the public health essay by audience segment and by a national population estimate. Note: 1 = (entirely positive comments); 0 = (mixed, including both positive and negative comments); and -1 = (entirely negative comments).

**Figure 3:**
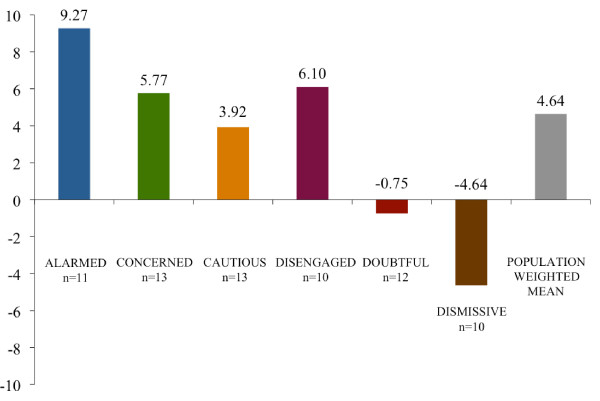
**Composite essay scores by segment**. Scores reflect respondent average values by segment for the difference between the number of times each of 18 sentences were marked "especially clear or helpful" and "especially confusing or unhelpful" with a full range of possible values between 18 and -18. The scores are adjusted for unequal numbers of respondents within each segment by re-weighting values to represent n = 10.

To test the between-segment differences in our dependent measures - overall reactions to the essay (i.e., valence) and composite sentence-specific reactions to the entire essay - we used the nonparametric Kruskal-Wallis test (see Figures 2, 3). To test if the median response to the essay on each dependent measure was greater than zero (i.e., a positive reaction) for our full sample, we used the Wilcoxon signed rank test. Lastly, for both dependent measures, we used the Wilcoxon signed rank test to test our hypothesis that five of the six segments (the Dismissive being the one exception) would respond positively to the essay; the null hypothesis was that the median score for each of the five segments did not differ from zero. The Wilcoxon signed rank test is appropriate for small sample sizes and non-normal distributions, both of which are the case for at least some segments in our data.

**Figure 4:**
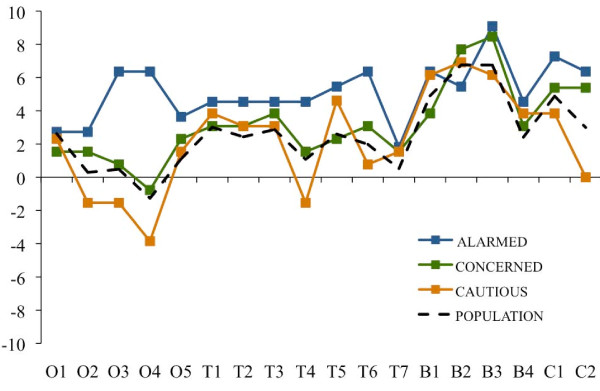
**Essay evaluations by sentence: Alarmed, Concerned and Cautious segments**. Sentence-specific evaluations of the public health essay by respondents in the Alarmed, Concerned and Cautious segments and by a national population estimate. Note: Scores reflect the difference between the number of times a sentence was marked as "especially clear or helpful" and the number of times it was marked as "especially confusing or unhelpful," adjusting for unequal numbers of respondents within each segment by re-weighting values to represent n = 10. Sentence abbreviations correspond to O = opening section (5 sentences); T = climate change health threat related section (7 sentences); B = mitigation-related policy actions and their health benefits (4 sentences); and C = concluding section (2 sentences). The national population estimate was created by weighting the values for each of the six segments according to their relative proportion of American adults.

**Figure 5:**
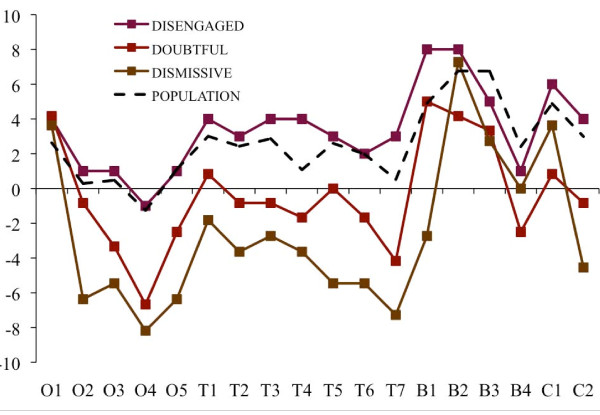
**Essay evaluations by sentence: Disengaged, Doubtful and Dismissive segments**. Sentence-specific evaluations of the public health essay by respondents in the Disengaged, Doubtful and Dismissive segments and by a national population estimate. Note: Scores reflect the difference between the number of times within a sentence was marked as "especially clear or helpful" and the number of times it was marked as "especially confusing or unhelpful," adjusting for unequal numbers of respondents within each segment by re-weighting values to represent n = 10. Sentence abbreviations correspond to O = opening section (5 sentences); T = climate change health threat related section (7 sentences); B = mitigation-related policy actions and their health benefits (4 sentences); and C = concluding section (2 sentences). The national population estimate was created by weighting the values for each of the six segments according to their relative proportion of American adults.

**Figure 6:**
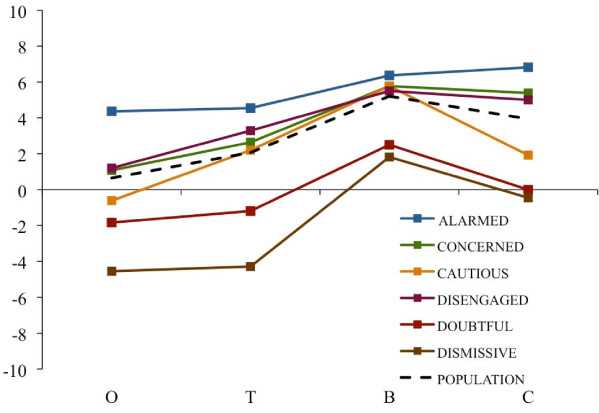
**Essay evaluations by section (opening, threat, benefits, closing)**. Average section-specific evaluations of the public health essay by respondents in each of the six audience segments and by a national population estimate. Note: Scores reflect the difference between the number of sentences within each section marked by a respondent as "especially clear or helpful" and those marked as "especially confusing or unhelpful" with those values averaged across the number of sentences per section and rescaled by a factor of 10. Section abbreviations correspond to O = opening section (5 sentences); T = climate change health threat related section (7 sentences); B = mitigation-related policy actions and their health benefits (4 sentences); and C = concluding section (2 sentences). The national population estimate was created by weighting the mean values for each of the six segments according to their relative proportion of American adults.

Post-hoc - after examining the visualized data (see Figures 4, 5 and 6) - we decided to test for two possible main effects in the data. To examine the possibility that the essay's later focus on the public health benefits of mitigation-related policy actions was seen by respondents as clearer and more useful than the essay's earlier focus on public health-related threats, we calculated the difference between the re-scaled (by a factor of 10) average response to both the benefit and the threat sections and then used the Wilcoxon signed rank test to test, by segment, whether the median of these differences was greater than zero. We then evaluated the overall main effect of the essay - across all segments - using the weighted t-test on the differences with weights corresponding to the frequencies of the segments in the population.

Lastly, to examine for the possibility that the concluding framing section of the essay was perceived by respondents as clearer and more useful than the opening framing section, we calculated the difference between the re-scaled average response to both the opening and the concluding sections and then used the Wilcoxon signed rank test to test, by segment, whether the median of these differences was greater than zero. We then evaluated the overall main effect - across all segments - using the weighted t-test on the differences with weights corresponding to the frequencies of the segments in the population.

## Results

### Overall Sample Response and Between-Group Differences

The results of non-parametric Kruskal-Wallis tests indicate that there are significant between-group differences for both dependent measures: valence (*p *= .001)and the composite sentence-specific score (*p *< .0001). For the overall sample, the Wilcoxon signed rank tests indicated a positive response on the sentence-specific composite score (*p *< .001) but not on the valence score (*p *= .12). The average valence scores - on a scale of 1 to -1 - spanned from .55 (Alarmed) to -.7 (Dismissive) (see Figure 2). The average sentence-specific composite scores - on a scale of 18 to -18 - ranged from 9.27 (Alarmed) to -4.64 (Dismissive) (see Figure 3).

### Hypothesis Test

The Wilcoxon signed rank tests indicated only partial support for our hypothesis. Using valence as the dependent measure, the null hypothesis *can be rejected *only for the Alarmed (*p *= .04) and Concerned (*p *= .02) segments, but not for the Cautious (*p *= .50), Disengaged (*p *= .36) or Doubtful segments (*p *= .50). Using the composite sentence-specific score as the dependent measure, the null hypothesis *can be rejected *for the Alarmed (*p *= .001), Concerned (*p *< .01) and Cautious (*p *= .01) segments, and marginally rejected for the Disengaged segment (*p *= .06), but not for the Doubtful segment (*p *= .61) segment.

In sum, there was clear evidence that the Alarmed and Concerned segments responded positively to the public health essay, and mixed evidence that the Cautious and Disengaged responded positively. There was no evidence that the Doubtful responded positively. It is worthy of note, however, that all six segments agreed with the essay's opening frame device (O1) that "good health is a great blessing," suggesting that human health and wellbeing is a widely shared value.

Table 3 summarizes the thematic content of the statements made by respondents when they were asked to discuss their general reactions to the public health essay. Across segments, not surprisingly, a substantial proportion of comments focused on the presentation of evidence or the stylistic tone of the essay. For the Alarmed and Concerned segments, roughly a third of their statements reflected personal agreement with the essay. In contrast, among the Dismissive, roughly a third of their statements characterized the essay as biased or alarmist. Relative to other possible reactions, substantial proportions of the statements made by the Concerned (18%), Cautious (19%), Disengaged (13%); and Doubtful (16%) indicated that the essay was informative and/or thought provoking.

**Table 3:** Distribution of Themes Expressed in Reaction to the Public Health Essay.

	Alarmed (n = 37) %	Concerned (n = 33) %	Cautious (n = 32) %	Disengaged (n = 32) %	Doubtful (n = 31) %	Dismissive (n = 28) %	Weighted population mean %
Critique of Proof/Style	22	18	34	28	26	21	24

Reflects My POV	32	33	9	22	10	11	23

Informative	5	18	19	13	16	4	14

Biased	0	9	9	6	16	32	10

Negative	8	6	6	3	3	11	6

Prescriptive	5	0	6	9	13	4	5

Credible	5	6	0	0	0	0	3

Too U.S. Centric	0	0	9	0	0	0	2

NA	22	9	6	19	16	18	13

### Benefit versus Threat Statements

The Wilcoxon signed rank tests used to compare segments on the perceived clarity and helpfulness of the threat statements in the first part of the essay against the health benefits of mitigation-related policy actions in the second part of the essay showed a significant main effect (*p *≤ .05) for all segments except the Alarmed (*p *= .17). The Dismissive segment showed the largest difference between the sections of the essay (6.10), followed by the Doubtful (3.69), the Cautious (3.57), the Concerned (3.13), and the Disengaged (2.12). Using a weighted t-test, the estimated gain from the Threat to Benefits sections across all segments was 3.17 (*p *< .0001), with a 95% confidence interval of 1.85 to 4.49. In short, the health benefits associated with mitigation-related policy actions were seen as clearer and more useful than the preceding threat statements in the essay.

Also worthy of note, as Figures 4 and 5 indicate, is that all six segments reacted positively to the following statements focusing on specific mitigation-related policy actions that lead to human health benefits:

"Taking actions to limit global warming - by making our energy sources cleaner and our cars and appliances more efficient, by making our cities and towns friendlier to trains, buses, and bikers and walkers, and by improving the quality and safety of our food - will improve the health of almost every American."

"Cleaner energy sources and more efficient use of energy will lead to healthier air for children and adults to breathe."

"Improving the design of our cities and towns in ways that make it easier to get around on foot, by bike and on mass transit will reduce the number of cars and help people become more physically active, lose weight."

Conversely, respondents in all segments responded less positively to the statement:

"Increasing our consumption of fruits and vegetables, and reducing our intake of meat - especially beef - will help people maintain a healthy weight, will help prevent heart disease and cancer, and will play an important role in limiting global warming."

### Opening versus Concluding Framing Statements

The Wilcoxon signed rank test used to compare segments on their reactions to the opening versus concluding framing statements for each segment showed a significant or marginally significant main effect in the Alarmed (*p *= .07), Concerned (*p *< .01), Cautious (*p *= .05), Disengaged (*p *= .03) and Dismissive (*p *< .01) segments; the trend was not significant in the Doubtful (*p *= .14) segment. The largest differences were seen in the Concerned segment (4.31), followed by the Dismissive (4.09), Disengaged (3.8), Cautious (2.54) and the Alarmed segment (2.45). Again using a weighted t-test, the estimated increase from the Opening to Concluding sections across all segments was 3.30 (*p *< .0001), with a 95% confidence interval of 2.14 to 4.47.

## Discussion

On the whole, people who read our public health-framed essay about climate change reacted positively to the information. People in the Alarmed and the Concerned segments demonstrated consistent positive response to the information, while people in the Cautious, Disengaged, and Doubtful segments were less consistent. Although we did not treat it as a dependent measure per se, many of the respondents in all five segments made open-ended comments about the essay that demonstrated a positive engagement with the material. For example, nearly half (44%) of the comments made by the Disengaged segment indicated that the essay reflected their personal point of view, was informative or thought-provoking, or offered valuable prescriptive information on how to take action relative to the climate problem. Similarly, 39% of the comments made by respondents in the Doubtful segment reflected one of these three themes. Moreover, the ascending sentence-specific evaluations between the opening and concluding sections of the essay, for the sample overall and for all of the segments (excluding the Dismissive), suggest that the value of the public health frame may not be immediate, but rather may manifest more fully after people have had time to consider the evidence, especially when this evidence is presented with specific mitigation-related policy actions that are likely to have human health benefits.

One of the most intriguing findings in the study - albeit not definitive due to the order effect of the information in the essay - is the robustness of the response across all six segments to information about the health benefits of taking action to address global warming.

Overall, we interpret these collective findings as providing partial support for our hypothesis that information about climate change framed in ways that encourage people to consider its human health context provides many Americans with a useful and engaging new frame of reference and that this new interpretation may broaden the personal significance and relevance of the issue. Our methods were exploratory, however, and additional research on this question is needed. To that end, we are further analyzing the data already collected to determine more systematically which specific ideas are most and also least resonant with members of each segment. We are also planning an experimental test of climate education material framed in various ways, including a public health frame. Additional research is needed to determine if these findings generalize across nations and other populations.

In the U.S., these findings are especially relevant given the "issue fatigue" that appears to be developing with regard to climate change among at least certain segments of the American public [26]. Recent public opinion polls in the U.S. have shown a marked decline in the proportion of adults who are worried about global warming, and even relative to the proportion who are convinced that global warming is happening [27-29]. The public health voice may offer an important hedge against such issue fatigue.

Suggesting a novel frame for climate change - i.e., a frame that people had not previously considered - is potentially useful when it helps people understand the issue more clearly by providing additional personal and societal relevance [30,31]. Re-defining climate change in public health terms should help people make connections to already familiar problems such as asthma, allergies, and infectious diseases experienced in their communities, while shifting the visualization of the issue away from remote Arctic regions, and distant peoples and animals. In the process, giving climate change a public health focus suggests that there is a need to both mitigate (i.e. reduce greenhouse gas emissions) and adapt to the problem (i.e. protect communities and people from current and future health related impacts). The frame also presents the opportunity to involve additional trusted communication partners on the issue, notably public health experts and local community leaders [13].

## Conclusions

In conclusion, we believe that the public health community has an important perspective to share about climate change, a perspective that potentially offers the public a more salient way to comprehend an issue that has proven deeply difficult for many people to fully comprehend. Moreover, the public health perspective offers a vision of a better, healthier future - not just a vision of environmental disaster averted, and it focuses on a range of possible policy actions that offer local as well as global benefits. Many leading experts in climate change communication, including the present authors, have suggested that a positive vision for the future and a localization of the issue is precisely what has been missing from the public dialogue on climate change thus far [13,22,32].

Not all aspects of the public health implications, however, may be engaging. Certain key recommendations, such as eating less meat, tended to elicit counter-arguments among people in many of the segments in our research. Our research provides clues about specific public health messages that might not be helpful, and suggests the need in future research to look carefully for examples or associations that trigger counter-arguments and negative reactions.

There is an urgent need for the public health community to successfully educate the public and policy makers about the serious human health implications of climate change, and to engage those publics in appropriate preventive and adaptive responses. As a point of strategy, however, our findings may suggest that continuing to communicate about the *problem of climate change *is not likely to generate wider public engagement. Instead public health voices may be wise to focus their communication on the *solutions and the many co-benefits *that matter most to people.

## Competing interests

The authors declare that they have no competing interests.

## Authors' contributions

EWM and MN developed the research question, participated in all aspects of the research, and wrote the first draft of the paper. PB coded and conducted preliminary data analysis. KA managed the data collection, conducted data analysis and prepared all figures and tables. GD conducted the final data analysis. All authors contributed to the final draft of the paper.

## Appendix 1

### Global Warming is a Threat to Peoples' Health & Wellbeing

Most people agree with the sentiment that "good health is a great blessing." Although not yet widely known, global warming poses a very real threat to the health and wellbeing of Americans and other people around the world. Experts at the World Health Organization say that global warming is already leading to an increase in the rate of some diseases and is causing many deaths. If our government and other governments around the world do not soon take steps to limit global warming, a growing number of people in the United States will likely be harmed and killed. Conversely, if our government does take steps to limit global warming, our health and wellbeing will likely improve in a number of important ways.

### Our health will suffer if we don't take action

Global warming can harm people both directly and indirectly. Directly, global warming causes more extreme weather patterns including more frequent heat waves, more violent storms, and rising sea-levels - all of which can lead to people being harmed or killed. Indirectly, global warming harms the quality of our water, air and food, and our ecosystems, all of which can lead to increasing rates of disease and death. If we do not act now to limit global warming, experts at the U.S. Centers for Disease Control and Prevention say that global warming will harm people in every region of the United States. As a result of the poor air quality caused by global warming, children will become more likely to develop asthma, and the asthma they suffer from will be more severe; adults who have heart and lung diseases will become more likely to be hospitalized or die from their illness. An increasing number of extreme heat waves, floods, storms, fires and droughts caused by the changes in our climate will lead to more people being injured or killed. New infectious diseases (such as West Nile Virus) and old infectious diseases that we had previously eradicated from the United States (such as malaria and Dengue Fever) are likely to become an increasing problem for us as our climate warms.

### Our health will benefit if we do take action

According to a recent study published in the medical journal Lancet, taking actions to limit global warming - by making our energy sources cleaner and our cars and appliances more efficient, by making our cities and towns friendlier to trains, buses, and bikers and walkers, and by improving the quality and safety of our food - will improve the health of almost every American. Cleaner energy sources and more efficient use of energy will lead to healthier air for children and adults to breathe. Improving the design of our cities and towns in ways that make it easier and safer to get around on foot, by bike and on mass transit will reduce the number of cars on our roads and will help people become more physically active and lose weight. Increasing our consumption of fruits and vegetables, and reducing our intake of meat - especially beef - will help people maintain a healthy weight, will help prevent heart disease and cancer, and will play an important role in limiting global warming.

### Conclusion

Peoples' health is dependent on the health of the environment in which we live. Global warming offers America an opportunity to make choices that are healthier for us, and for our climate.

## Pre-publication history

The pre-publication history for this paper can be accessed here:

http://www.biomedcentral.com/1471-2458/10/299/prepub

## References

[B1] Karl TR Melillo JM Peterson TC (Eds) Global climate change impacts in the United States 2009 Cambridge University Press

[B2] IPCC Parry ML, Canziani OF, Palutikof JP, van der Linden PJ, Hanson CE Climate change 2007: impacts, adaptation and vulnerability. Contribution of Working Group II to the Fourth Assessment Report of the Intergovernmental Panel on Climate Change 2007 Cambridge University Press

[B3] Frumkin H McMichael AJ Climate change and public health: thinking, acting and communicating American Journal of Preventive Medicine 2008 35 5 403 410 10.1016/j.amepre.2008.08.019 18929964

[B4] Jay M Marmot MG Health and climate change: will a global commitment be made at the UN climate change conference in December? BMJ 2009 339 645 646 10.1136/bmj.b3669

[B5] Environmental Protection Agency Endangerment and cause or contribute findings for greenhouse gases under Section 202(a) of the Clean Air Act Federal Register 2009 74 239 66495 66546 http://www.epa.gov/climatechange/endangerment/downloads/Federal_Register-EPA-HQ-OAR-2009-0171-Dec.15-09.pdf

[B6] McMichael AJ Woodruff RE Hales S Climate change and human health: present and future risks Lancet 2006 367 859 69 10.1016/S0140-6736(06)68079-3 16530580

[B7] Maibach EW Roser-Renouf C Leiserowitz A Global warming's Six Americas 2009: an audience segmentation analysis 2009 http://climatechange.gmu.edu 10.1371/journal.pone.0017571 PMC3053362 21423743

[B8] Berry P Clarke K Pajot M Hutton D Verret M The role of risk perception and health communication in adapting to the health impacts of climate change in Canada Natural Resources Canada 2009 Ottawa, Canada

[B9] DeBono R Maltese public perceptions on climate change and health MPH thesis 2009 University of Malta, Faculty of Medicine and Surgery

[B10] Leiserowitz A American risk perceptions: is climate change dangerous? Risk Anal 2005 25 1433 42 10.1111/j.1540-6261.2005.00690.x 16506973

[B11] Gamson WA Croteau D Hoynes W Sasson T Media images and the social construction of reality Annual Review of Sociology 1992 18 373 93 10.1146/annurev.so.18.080192.002105

[B12] Price V Nir L Capella JN Framing public discussion of gay civil unions Public Opinion Quarterly 2005 69 2 179 212 10.1093/poq/nfi014

[B13] Nisbet MC Communicating climate change: why frames matter to public engagement Environment 2009 51 2 514 518

[B14] Ockwell D Whitmarsh L O'Neill S Reorienting climate change communication for effective mitigation: forcing people to be green or fostering grass roots engagement? Science Communication 2009 30 3 305 327 10.1177/1075547008328969

[B15] Balbus J Ebi K Finzer L Malina C Chadwick A McBride D Chuk M Maibach E Are we ready?: preparing for the public health challenges of climate change Environmental Defense Fund 2008

[B16] Maibach EW Chadwick A McBride D Chuk M Ebi K Balbus J Climate change and local public health in the United States: Preparedness, programs and perceptions of local public health department directors PLoS ONE 2008 3 7 10.1371/journal.pone.0002838 18665266 PMC2474970

[B17] Association of State and Territorial Health Officials Climate change: a serious threat to public health 2009 http://www.astho.org/Search.aspx?s=climate%20change

[B18] World Health Organization WHO workplan on climate change and health 2009 http://www.who.int/globalchange/wha_plans_objectives/en/index1.html

[B19] The Lancet 2007 370 9591 10.1016/S0140-6736(07)61431-7 17869622

[B20] The Lancet 2009 374 9707 10.1016/S0140-6736(09)62136-X 20109815

[B21] Frumkin H McMichael AJ Hess JJ (Eds) Climate change and the health of the public American Journal of Preventive Medicine 2008 35 5 10.1016/j.amepre.2008.08.031 18929963

[B22] Maibach EW Roser-Renouf C Leiserowitz A Communication and marketing as climate change-intervention assets: A public health perspective American Journal of Preventive Medicine 2008 35 5 488 500 10.1016/j.amepre.2008.08.016 18929975

[B23] U. S. Census Bureau Census Bureau Regions and Divisions with State FIPS Codes http://www.census.gov/geo/www/us_regdiv.pdf Retrieved April 21, 2010

[B24] Hayes AF Krippendorff K Answering the call for a standard reliability measure for coding data Communication Methods and Measures 2007 1 77 89

[B25] Krippendorff K Content Analysis: An Introduction to Its Methodology 1980 Newbury Park, CA: Sage

[B26] Kerr RA Amid worrisome signs of warming, 'climate fatigue' sets in Science 2009 326 926 928 10.1126/science.326.5955.926 19965491

[B27] ABC News/Washington Post Conservatives, Republicans move away from belief that the Earth is warming http://abcnews.go.com/images/PollingUnit/1096a7GlobalWarming.pdf

[B28] Pew Research Center for the People & the Press Fewer Americans see solid evidence of global warming http://people-press.org/report/556/global-warming

[B29] Gallup Increased number think global warming is "exaggerated." http://www.gallup.com/poll/116590/Increased-Number-Think-Global-Warming-Exaggerated.aspx

[B30] Price V Tewksbury D Barnett GA, Boster FJ News values and public opinion: a theoretical account of media priming and framing Progress in the Communication Sciences 1997 13 New York: Ablex 173 212

[B31] Scheufele DA Tewksbury D Framing, agenda setting and priming: the evolution of three media effects models Journal of Communication 2007 57 1 9 20

[B32] Moser SC Dilling L Creating a Climate for Change: Communicating Climate Change and Facilitating Social Change 2007 Cambridge, UK: Cambridge University Press

